# Modifying inhibitor specificity for homologous enzymes by machine learning

**DOI:** 10.1111/febs.70249

**Published:** 2025-09-05

**Authors:** Dor S. Gozlan, Reut Meiri, Gili Shapira, Matt Coban, Evette S. Radisky, Yaron Orenstein, Niv Papo

**Affiliations:** ^1^ Avram and Stella Goldstein‐Goren Department of Biotechnology Engineering Ben‐Gurion University of the Negev Beer‐Sheva Israel; ^2^ Department of Computer Science Bar‐Ilan University Ramat Gan Israel; ^3^ Department of Cancer Biology Mayo Clinic Comprehensive Cancer Center Jacksonville FL USA; ^4^ The Mina and Everard Goodman Faculty of Life Sciences Bar‐Ilan University Ramat Gan Israel; ^5^ National Institute of Biotechnology in the Negev Ben‐Gurion University of the Negev Beer‐Sheva Israel

**Keywords:** deep mutational scanning, matrix metalloproteinases, neural networks, protein engineering, protein–protein interactions

## Abstract

Selective inhibitors are essential for targeted therapeutics and for probing enzyme functions in various biological systems. The two main challenges in identifying such protein‐based inhibitors lie in the extensive experimental effort required, including the generation of large libraries, and in tailoring the selectivity of inhibitors to enzymes with homologous structures. To address these challenges, machine learning (ML) is being used to improve protein design by training on targeted libraries and identifying key interface mutations that enhance affinity and specificity. However, such ML‐based methods are limited by inaccurate energy calculations and difficulties in predicting the structural impacts of multiple mutations. Here, we present an ML‐based method that leverages HTS data to streamline the design of selective protease inhibitors. To demonstrate its utility, we applied our new method to find inhibitors of matrix metalloproteinases (MMPs), a family of homologous proteases involved in both physiological and pathological processes. By training ML models on binding data for three MMPs (MMP‐1, MMP‐3, and MMP‐9), we successfully designed a novel N‐TIMP2 variant with a differential specificity profile, namely, high affinity for MMP‐9, moderate affinity for MMP‐3, and low affinity for MMP‐1. Our experimental validation showed that this novel variant exhibited a significant specificity shift and enhanced selectivity compared with wild‐type N‐TIMP2. Through molecular modeling and energy minimization, we obtained structural insights into the variant's enhanced selectivity. Our findings highlight the power of ML‐based methods to reduce experimental workloads, facilitate the rational design of selective inhibitors, and advance the understanding of specific inhibitor–enzyme interactions in homologous enzyme systems.

## Graphical abstract

We present a machine learning method for the design of selective enzyme inhibitors, reducing experimental effort thus accelerating the development of targeted therapeutics. Trained on existing data for three related matrix metalloproteinases (MMPs), our method successfully designed a novel inhibitor variant with enhanced selectivity for one MMP over the others. Using computer simulations, we gained a structural understanding of how the new inhibitor works and why it is more selective.

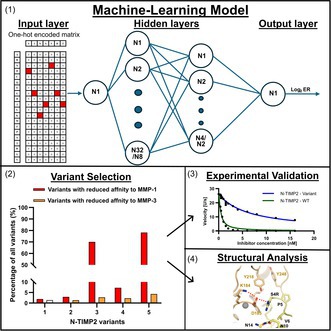

**Abbreviations**
DMS: deep mutational scanning
ER: enrichment ratio
HTS: high‐throughput sequencing
ML: machine learning
MMP: matrix metalloproteinase
NF: normalized frequency
N‐TIMP2: N‐terminal inhibitory domain of the tissue inhibitor of MMP‐2

## Introduction

Countless biological processes, including metabolism, signal transduction, and tissue remodeling, to name but a few, are regulated by enzymes. Imbalances in enzyme activity can thus lead to severe disorders, including cancer, neurodegeneration, and inflammation [1]. Thus, inhibitors serve as vital tools to control enzyme activity—both for therapeutic intervention and for the study of enzyme functions. However, despite the research attention devoted to their study, the design of inhibitors with precise specificity for a particular enzyme remains challenging, particularly for enzymes that coexist alongside other enzymes with homologous structures and/or overlapping mechanisms [2, 3].

Traditional techniques for finding specific protein‐based enzyme inhibitors have obvious drawbacks, as they often involve the tedious process of altering individual residues and assessing resultant changes in binding affinity [4]. Recent years have nonetheless seen notable advances in the design and production of inhibitors with tailored specificity for clinically relevant enzymes, but several drawbacks still remain [5]. In particular, the widely used combinatorial method, also referred to as directed evolution, entails the construction of extensive libraries of protein variants with mutations at random positions. Variants exhibiting the desired binding properties are then selected, and high‐throughput sequencing (HTS) is used to determine their sequences. This ‘non‐rational’ approach yields high‐affinity, but not necessarily selective, binders, but is nonetheless employed for a wide range of end uses, including affinity enhancement [6] and the design and production of high‐affinity inhibitors from antibodies [7], natural protein effectors [8, 9], and other protein domains [10, 11, 12, 13, 14]. The few studies that were indeed aimed at producing selective binders were restricted to enhancing the discrimination between two—but not more—target enzymes possessing distinct binding epitopes [15, 16, 17], whereas in reality, broad‐spectrum enzyme binders may interact with more than two potential target enzymes with binding epitopes exhibiting significant sequence homology and structural similarity.

Another drawback of directed evolution studies is the number of variants that can be robustly tested using such approaches—typically limited to several millions of variants out of billions of possibilities. Consequently, only a small portion of the protein sequence space (which grows exponentially with the number of mutated positions) can be explored. Thus, in binding selection experiments, only a few positions in a protein can be randomized to all 20 amino acids. However, the number of protein residues influencing binding affinity, and particularly those necessary for achieving binding specificity, whether through direct contacts or allosteric effects, is often substantial, particularly if the target proteases share similar structures [18].

To address the above‐described drawbacks, computational protein design has been employed to design targeted libraries of protein inhibitors. This methodology involves pinpointing positions on the inhibitor‐enzyme interface where mutations hold the greatest potential for enhancing affinity and specificity, while minimizing the risk of compromising the inhibitor's structure [19]. However, this methodology is limited due to inaccurate energy functions in computing binding energetics, which may overlook the correct conformations of the mutated residues in the binding interface. Additionally, it necessitates comprehensive knowledge, often unavailable, of the structure and function of the inhibitor‐enzyme complex [20]. Furthermore, while computational protein design can accurately predict affinity‐boosting protein variants with single mutations, it is less successful in enhancing the affinity of variants with multiple mutations; the effects of such mutations on protein structure are more difficult to predict, although they are crucial for conferring binding specificity, especially for target enzymes with similar structures [21].

As a model scenario for addressing the above challenges, we chose to develop a machine learning (ML) method for designing specific protein‐based inhibitors of the catalytic domain of matrix metalloproteinases (MMPs), a family of homologous proteases involved in both physiological and pathological processes. The inhibitors we developed are variants of N‐TIMP2, the N‐terminal inhibitory domain of tissue inhibitor of metalloproteinases 2 (TIMP2), which prevents the enzymatic activity of MMPs by binding to their active site in the catalytic domain (MMP_CAT_). The highly conserved active site of all MMPs contains a catalytic zinc ion coordinated to three histidine residues and a catalytic glutamate residue [22]. We focused on three human MMPs representing distinct functional categories: collagenases (MMP‐1), stromelysins (MMP‐3), and gelatinases (MMP‐9). Despite the ~ 45% sequence identity and close structural similarity of their catalytic domains, these MMPs exhibit variable binding affinities for N‐TIMP2, spanning a range of two orders of magnitude [23, 24]. This broad‐spectrum of affinities makes N‐TIMP2 an ideal model for evaluating our approach, by allowing us to intentionally engineer switches in specificity. Furthermore, previous studies have shown that multiple positions at the N‐TIMP2 binding interface can be mutated with minimal disruption of protein stability to shape selectivity [21, 24, 25]. Consequently, maximal coverage of this large mutation space is required to optimize binding selectivity for the three MMPs. An illustrative example is the recent publication of Rotenberg *et al*. [26] that described the screening of N‐TIMP2 variants with multiple mutations for high affinity to MMP‐9. Variants with improved selectivity were identified by chance rather than through a directed approach to engineer specificity, highlighting the importance of developing methods that can target specificity directly rather than relying on chance.

In this study, we exploited HTS data derived from affinity screening of N‐TIMP2 libraries against MMP‐1_CAT_, MMP‐3_CAT_, and MMP‐9_CAT_ (hereafter referred to as MMP‐1, MMP‐3, and MMP‐9), previously generated in our lab. We trained an ML model, based on a supervised learning methodology, to predict the binding affinity between N‐TIMP2 variants and these three MMPs. Our goal was to design a novel N‐TIMP2 variant with binding superiority for MMP‐9 and discriminative binding between MMP‐1 and MMP‐3. We identified high‐affinity N‐TIMP2 variants for MMP‐9 using our previously published model [27] and trained new affinity models for N‐TIMP2/MMP‐1 and N‐TIMP2/MMP‐3 based on our HTS datasets. We selected a single N‐TIMP2 variant, designated N‐TIMP2_MUT_, and validated it by inhibition assays against the three MMPs. Finally, we provided a potential structural explanation for the selectivity of N‐TIMP2_MUT_ through molecular modeling and energy minimization tests.

## Results

### Predicting an N‐TIMP2 variant with superior binding affinity for MMP‐9 vs. MMP‐1 and MMP‐3

Wild‐type N‐TIMP2 (N‐TIMP2_WT_) has similar affinities for MMP‐1 and MMP‐9 but a 10‐fold lower affinity for MMP‐3, thereby making it an ideal candidate for engineering selective binding [26]. To design a novel N‐TIMP2 variant with differential affinity for MMPs, namely, high affinity for MMP‐9, moderate affinity for MMP‐3, and low affinity for MMP‐1, we trained three ML models to predict the affinity between a given N‐TIMP2 variant and an MMP. These models, namely, N‐TIMP2/MMP‐1, N‐TIMP2/MMP‐3, and N‐TIMP2/MMP‐9, were trained on HTS data derived from an N‐TIMP2 library with mutations in seven key positions (i.e., 4, 35, 38, 68, 71, 97, and 99, PDB ID: 1BUV). These positions were selected due to their known importance for MMP binding and inhibition (i.e., their proximity, within 4 Å, to the MMP interface and to the catalytic zinc in the complex structure) and their structural tolerance to mutagenesis [23]. Specifically, we trained the models to predict the log_2_ enrichment ratio (ER)—a value quantifying the relative enrichment of a variant in one library fraction compared to another, that is, a sorted library fraction compared to an unsorted, naïve library. By using these models to predict binding affinities, we designed a novel variant with predicted high affinity for MMP‐9, moderate affinity for MMP‐3, and low affinity for MMP‐1.

To predict N‐TIMP2 variants with high affinity for MMP‐9, we utilized our previously developed N‐TIMP2/MMP‐9 model [27], which has a high predictive performance for variants with single mutations. While a strong improvement in affinity to a single target may be obtained with no more than one mutation, selectivity in binding may require N‐TIMP2 variants with multiple mutations. Accordingly, we selected five N‐TIMP2 variants with mutations at all seven interface positions that had the strongest predicted log_2_ ER values (Table 1).

**Table 1:** Wild‐type and selected variants with highest predicted binding affinity (predicted log_2_ ER) to MMP‐9.

Mutant	Amino acid at position							Predicted log_2_ ER
	4	35	38	68	71	97	99	
N‐TIMP2_WT_ ^a^	S	I	N	S	V	H	T	−0.218
N‐TIMP2_M1_	R	T	D	W	W	I	Q	12.561
N‐TIMP2_M2_	R	M	D	W	W	I	Q	12.319
N‐TIMP2_M3_	R	T	D	W	W	I	D	12.312
N‐TIMP2_M4_	R	F	D	W	W	I	Q	12.118
N‐TIMP2_M5_	R	F	D	W	W	I	D	12.107

^a^ N‐TIMP2_WT_ is the wild‐type version (i.e., without mutations).

To select from these five variants an N‐TIMP2 variant with the desired selectivity properties, we developed N‐TIMP2/MMP‐1 and N‐TIMP2/MMP‐3 models to be used in conjunction with our previously developed N‐TIMP2/MMP‐9 model; for this purpose, we adopted the methodological framework previously developed for the N‐TIMP2/MMP‐9 model [27]. The training HTS datasets were generated from an N‐TIMP2_WT_ saturation mutagenesis library with single mutations at the seven key positions. That library was sorted into library fractions (designated MMP‐1_Low_, MMP‐3_Low_, MMP‐1_High_, and MMP‐3_High_) having either low or high affinities to MMP‐1 and MMP‐3 (Table 2).

**Table 2:** Read counts of variants categorized by the number of mutations in the five libraries.

Library fraction	Number of mutations^a^						Total variants
	0	1	2	3	4	5	
Original library	1	131/140	3142/8.4 × 10^3^	1526/2.8 × 10^5^	81/5.6 × 10^6^	1/6.7 × 10^7^	4882/20^7^
MMP‐1_Low_	1	124	1250	104	0	0	1479
MMP‐1_High_	1	123	1727	82	0	0	1933
MMP‐3_Low_	1	127	1427	85	0	0	1640
MMP‐3_High_	1	131	1964	124	0	0	2220

^a^ The number of mutations vs. N‐TIMP2_WT_. The dataset lacked variants with six or seven mutations.

**Fig. 1:**
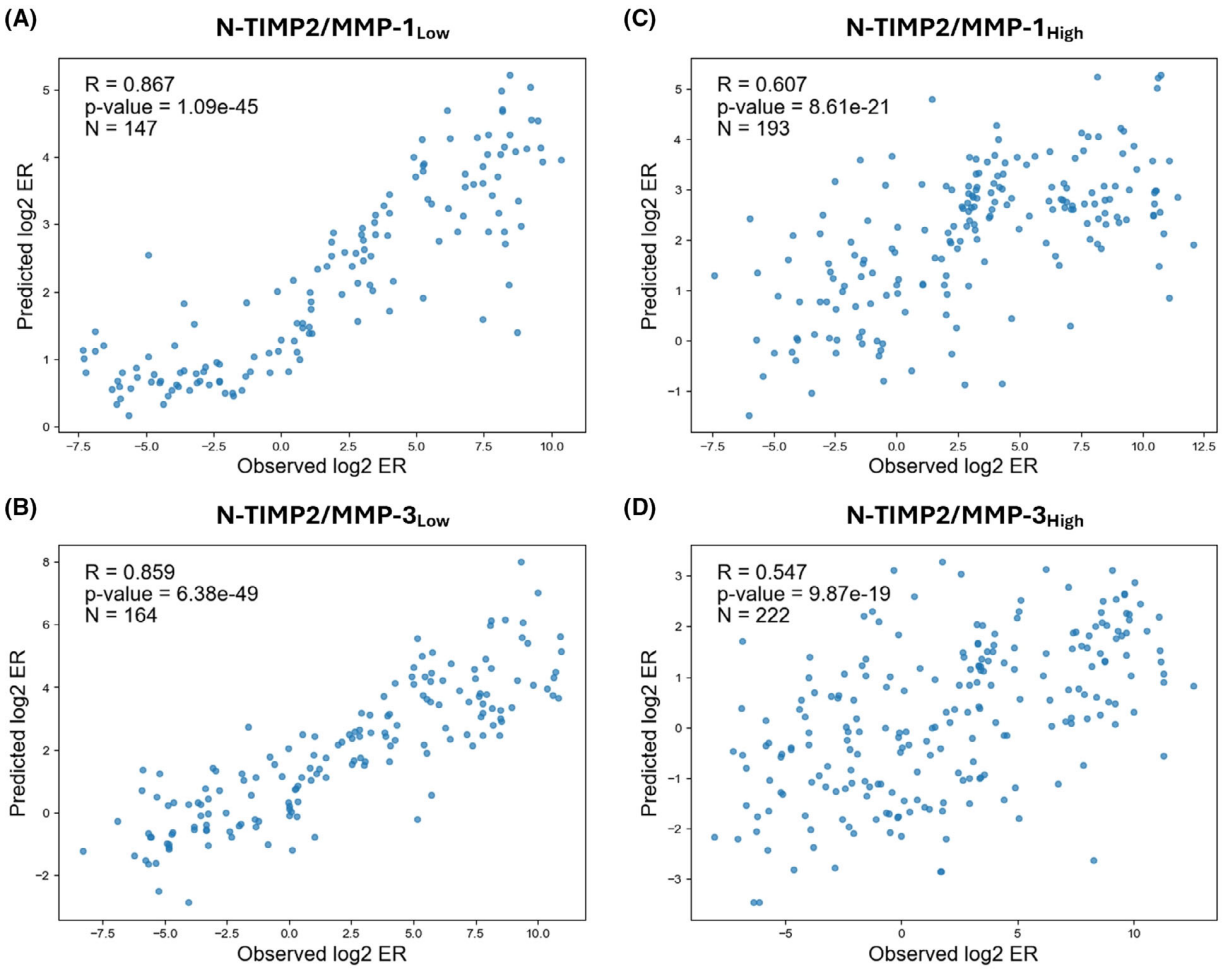
Evaluation of N‐TIMP2/MMP‐1_Low_, N‐TIMP2/MMP‐3_Low_, N‐TIMP2/MMP‐1_High_, and N‐TIMP2/MMP‐3_High_ models trained on low‐ and high‐affinity datasets, respectively. (A, B) Correlation between predicted and observed log_2_ enrichment ratio (ER) values for low‐affinity datasets of matrix metalloproteinase‐1 (MMP‐1) and MMP‐3. (C, D) Correlation for high‐affinity datasets of MMP‐1 and MMP‐3. Correlation coefficients (*R*), *p*‐values, and number of data points (*N*) are indicated on each panel. N‐TIMP2 refers to the N‐terminal inhibitory domain of tissue inhibitor of metalloproteinases‐2.

Our models trained on low‐affinity HTS data (N‐TIMP2/MMP‐1_Low_ and N‐TIMP2/MMP‐3_Low_) achieved higher performance on their respective test set than our models trained on high‐affinity HTS data (N‐TIMP2/MMP‐1_High_ and N‐TIMP2/MMP‐3_High_): Pearson correlations for the low‐affinity models of MMP‐1 and MMP‐3 were 0.867 and 0.859, respectively (Fig. 1A,B), while the correlations for the high‐affinity models were 0.607 and 0.547, respectively (Fig. 1C,D).

**Fig. 2:**
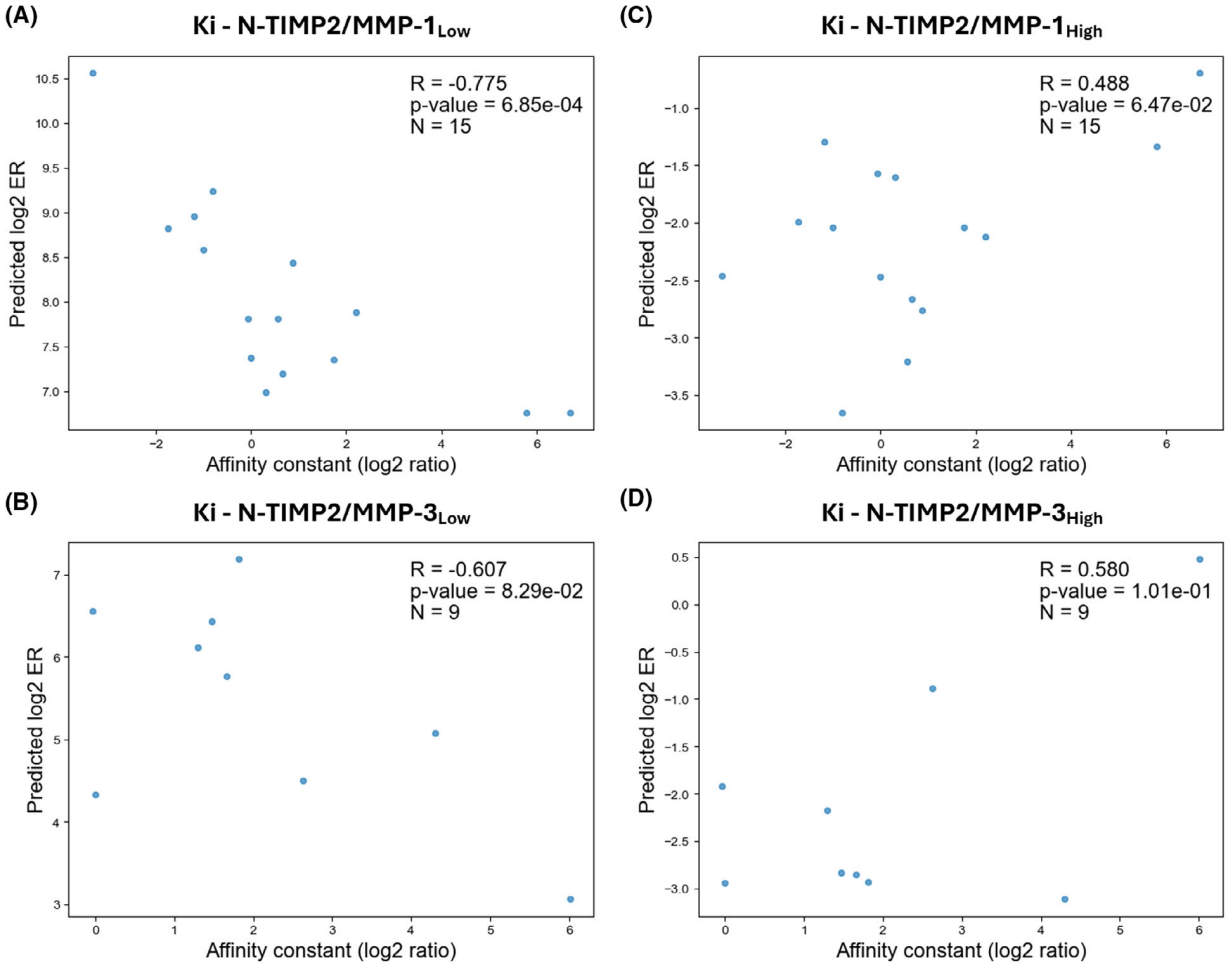
Independent validation of the four machine learning (ML) models using inhibition data for purified MMPs. Correlations between observed log_2_ enrichment ratio (ER) values and predicted log_2_ relative inhibition constant (*K*_*i*_) values for MMP‐1 (A, C) and MMP‐3 (B, D). Correlation coefficients (*R*), *P*‐values, and number of data points (*N*) are shown on each panel.

The above trend was similar to that for prediction of the inhibition constants (*K*_*i*_) of selected purified N‐TIMP2 variants (15 variants for MMP‐1 and 9 for MMP‐3), where the Pearson's correlations for the low‐affinity models were −0.775 (MMP‐1) and −0.607 (MMP‐3) (Fig. 2A,B) compared to 0.488 (MMP‐1) and 0.58 (MMP‐3) for the high‐affinity models (Fig. 2C,D). These findings demonstrate the superior predictive performance of the low‐affinity models, which we then used for the subsequent steps of the variant design process.

**Fig. 3:**
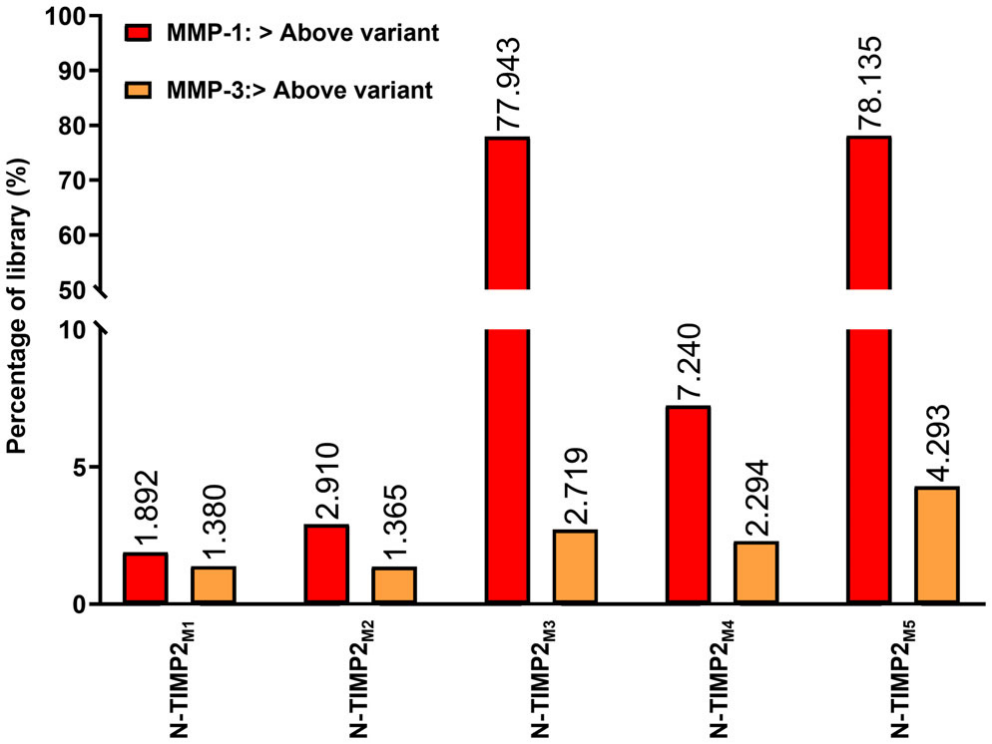
Affinity ranking of selected N‐TIMP2 variants in terms of binding to MMP‐1 and MMP‐3. The bar graph presents the percentage of N‐TIMP2 variants with predicted affinity greater than the affinity predicted for each selected variant (i.e., M1, M2, M3, M4, and M5, Table 1); the affinity ranking indicates the selectivity of the variants for MMP‐1 and MMP‐3.

Using the N‐TIMP2/MMP‐1_Low_ and N‐TIMP2/MMP‐3_Low_ models, we selected from the five variants with the highest affinity for MMP‐9 one variant that also exhibited moderate affinity for MMP‐3 and low affinity for MMP‐1, by applying the following procedure. We first predicted the log_2_ ER values for N‐TIMP2/MMP‐1_Low_ and N‐TIMP2/MMP‐3_Low_ models for all possible 20^7^ N‐TIMP2 variants and compared their values to the predictions for the five variants with the highest predicted affinity for MMP‐9. We then ranked the five variants, in terms of binding to MMP‐1 and MMP‐3, by calculating the percentage of library variants having higher log_2_ ER values than that of each of the five variants, demonstrating that less than 10% of library variants had lower affinity for MMP‐3 (Fig. 3). Among the five selected variants, N‐TIMP2_M3_ and N‐TIMP2_M5_ showed differential binding affinity between MMP‐1 and MMP‐3, making these two variants appropriate candidates for further investigation. We arbitrarily chose N‐TIMP2_M5_ and refer to it henceforth as N‐TIMP2_MUT_.

### N‐TIMP2_MUT_ exhibits differential inhibition potencies to MMP‐9, MMP‐3, and MMP‐1

**Fig. 4:**
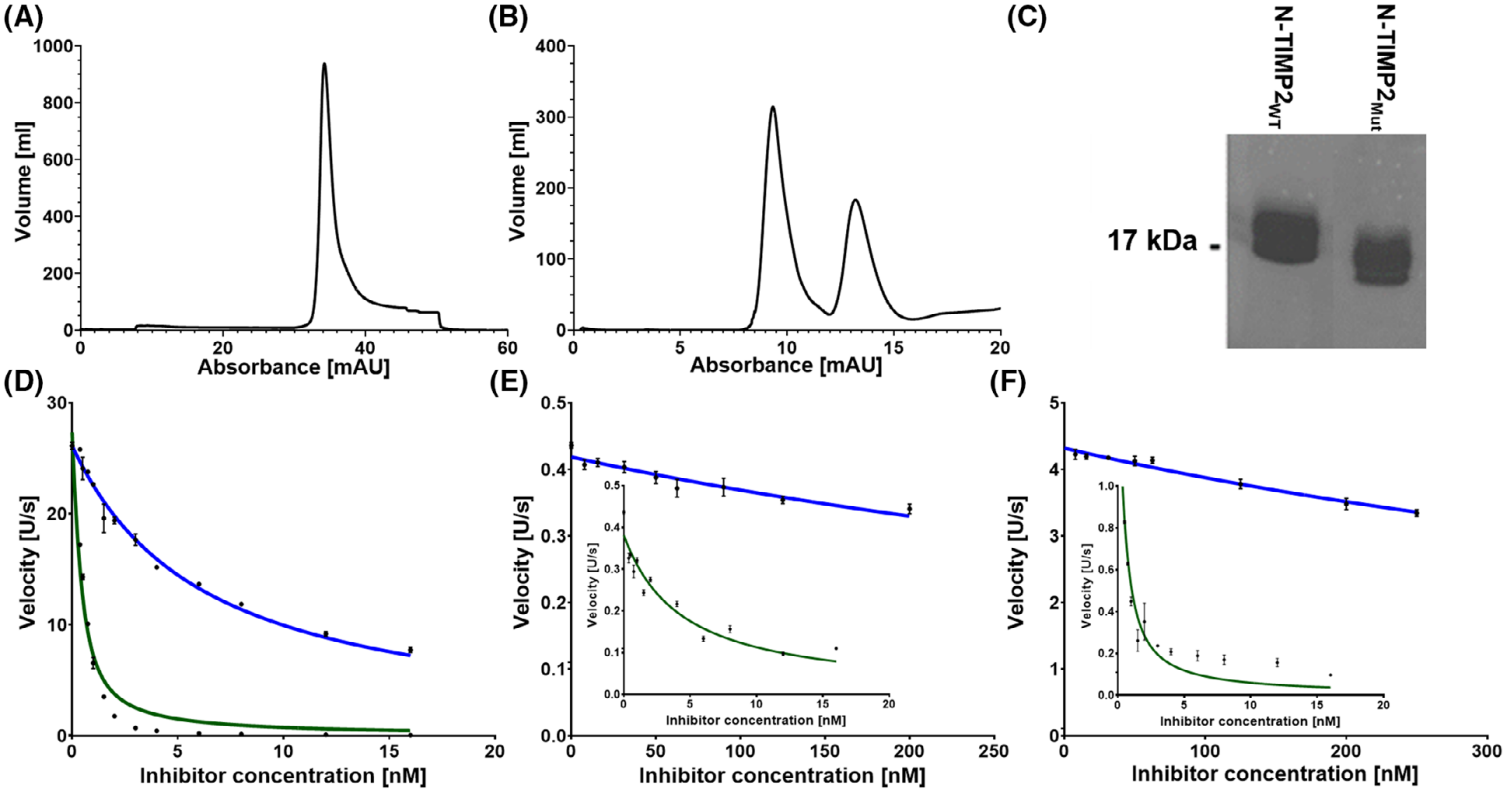
Purification and characterization of inhibition of N‐TIMP2_WT_ and N‐TIMP2_MUT_ proteins. (A, B) Purification of N‐TIMP2_MUT_ using affinity chromatography (nickel‐nitrilotriacetic acid, Ni‐NTA) and size‐exclusion chromatography (Superdex 75), respectively. (C) Analysis of purified N‐TIMP2_WT_ and N‐TIMP2_MUT_ by 15% sodium dodecyl sulfate–polyacrylamide gel electrophoresis (SDS/PAGE) under non‐reducing conditions. (D–F) Inhibition by N‐TIMP2_WT_ (dark green) and N‐TIMP2_MUT_ (blue) of the catalytic activity of MMP‐9, MMP‐3, and MMP‐1, respectively. We followed the cleavage in fluorescence units per seconds (U·s^−1^) of the MMP substrate Mca‐Pro‐Leu‐Gly‐Leu‐Dpa‐Ala‐Arg‐NH_2_·TFA [Mca = (7‐methoxycoumarin‐4‐yl)acetyl, Dpa = *N*‐3‐(2,4‐dinitrophenyl)‐l‐2,3‐diaminopropionyl and TFA = trifluoroacetic acid] over time, and determined the initial reaction velocities at each inhibitor concentration. To obtain the inhibition constants (*K*_*i*_), we fitted the data by multiple regression to Morrison's tight binding inhibition equation (Eqn 5; see Materials and methods). Data is presented as the average of three independent experiments; error bars represent standard deviation.

As the first step in examining the inhibition of MMP‐1, MMP‐3, and MMP‐9 by N‐TIMP2_WT_ or N‐TIMP2_MUT_, we expressed the N‐TIMP2 proteins and purified them in soluble form from the yeast *Pichia pastoris*, as previously described [25]. Briefly, we expressed the proteins from the pPICZαA vector that produces versions of the proteins with a native mature free N terminus, which is required for inhibitory activity, and C‐terminal His‐ and c‐Myc epitope tags. We purified the proteins by affinity chromatography, followed by size‐exclusion chromatography (Fig. 4A,B). We confirmed the sizes and purity of the variants by sodium dodecyl sulfate/polyacrylamide gel electrophoresis (SDS/PAGE) (Fig. 4C). To determine the binding affinities of purified N‐TIMP2_WT_ or N‐TIMP2_MUT_ to MMP‐1, MMP‐3, and MMP‐9, we performed enzyme kinetics experiments using a fluorogenic reporter substrate and determined the *K*_*i*_ values for each complex (Fig. 4D–F).

The *K*_*i*_ values for N‐TIMP2_WT_ in complex with MMP‐1, MMP‐3, and MMP‐9 were 0.11 ± 0.03, 1.38 ± 0.31, and 0.11 ± 0.01 nm, respectively—values that are consistent with those reported in previous studies [19, 21, 22, 23]. The *K*_*i*_ values of N‐TIMP2_MUT_ interacting with MMP‐1, MMP‐3, and MMP‐9 were 283 ± 45, 248.3 ± 65.6, and 2.341 ± 0.116 nm, respectively. These values indicate a significant enhancement in the selectivity of TIMP2_MUT_ (relative to TIMP2_WT_) for MMP‐9 versus MMP‐3 and MMP‐1, namely, N‐TIMP2_MUT_ binds to MMP‐9 ~106 and ~ 121 times more strongly than to MMP‐3 and MMP‐1, respectively, whereas N‐TIMP2_WT_ binds only ~ 13 and ~ 1 times stronger to MMP‐9 than to MMP‐3 and MMP‐1, respectively (Table 3). While enhancement in selectivity toward MMP‐9 was obtained for N‐TIMP2_MUT_ (relative to TIMP2_WT_), this enhancement was accompanied by a reduction in affinity for all three MMPs (by ~ 180‐, ~ 2572‐, and ~ 21‐fold for MMP‐3, MMP‐1 and MMP‐9, respectively).

**Table 3:** Inhibition constants (*K*_i_) of MMP‐1, MMP‐3, and MMP‐9 with N‐TIMP2 proteins.

N‐TIMP2	MMP‐9			MMP‐3			MMP‐1		
	$K_{i}^{\mathrm{app}}$ (nm)	*K*_*i*_ ^a^ (nm)	*K*_*i*_ ^b^ fold	$K_{i}^{\mathrm{app}}$ (nm)	*K*_*i*_ ^a^ (nm)	*K*_*i*_ ^b^ fold	$K_{i}^{\mathrm{app}}$ (nm)	*K*_*i*_ ^a^ (nm)	*K*_*i*_ ^b^ fold
N‐TIMP2_WT_	0.2766 ± 0.024	0.11 ± 0.01	1	4.125 ± 0.461	1.38 ± 0.31	13	0.367 ± 0.032	0.11 ± 0.03	1
N‐TIMP2_MUT_	6.036 ± 0.301	2.341 ± 0.116	1	742.0 ± 95.1	248.3 ± 65.6	106	872.4 ± 66.8(904) 953‐6372	283 ± 45	121

^a^ *K*_*i*_ and $K_{i}^{\mathrm{app}}$ values (nm) of the purified variants were obtained by fitting the experimental data to Morrison's tight binding equation (Eqn 5; see Materials and methods).

^b^ Fold change of *K*_*i*_ indicates the ratio between the *K*_*i*_ of N‐TIMP2 (either N‐TIMP2_WT_ or N‐TIMP2_MUT_) in complex with MMP‐3 (or MMP‐1) and the *K*_*i*_ of N‐TIMP2 in complex with MMP‐9

### Molecular modeling of the binding of N‐TIMP2_MUT_ to MMPs

**Fig. 5:**
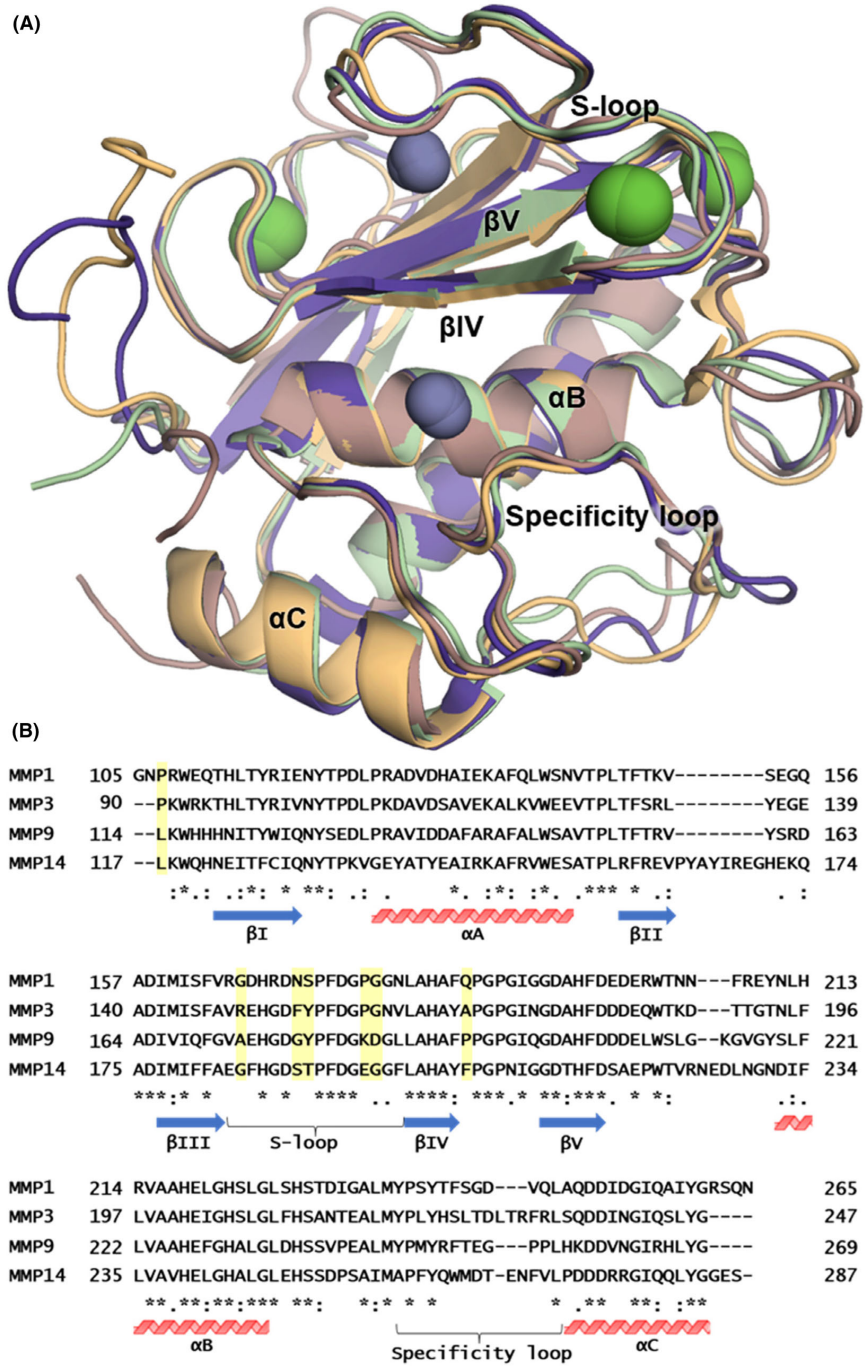
Structural and sequence comparison of MMPs. (A) Superposition of the catalytic domain crystal structures of MMP‐14 (PDB ID: 1BUV, *pink*), MMP‐9 (PDB ID: 4JIJ, *light orange*), MMP‐3 (PDB ID: 1UEA, *purple*), and MMP‐1 (PDB ID: 2J0T, *green*) reveals a common fold. N‐TIMP2 interacts with the catalytic zinc and substrate‐binding channel that crosses the enzyme horizontally above helix αB, interacting with the S‐loop and specificity loop. (B) Sequence alignment of the MMP catalytic domains identifies conserved and variable regions. Secondary structure elements and critical substrate‐binding loops are annotated below each sequence. Non‐conserved residues highlighted in yellow may contribute to the enhanced selectivity of N‐TIMP2_MUT_, as described in the text. The structural comparison was performed using pymol Molecular Graphics System (Version 2.5.2), with minimal modifications to the original structures (superposing, hiding waters, etc). The sequence alignment was carried out with clustal omega server (https://www.ebi.ac.uk/jdispatcher/msa/clustalo?stype=protein), and the graphic for this was manually constructed in PowerPoint. The sequences were obtained from UniProt with the following accession numbers: MMP‐1: P03956; MMP‐3: P08254; MMP‐9: P14780; MMP‐14: P50281.

Crystal structures reveal that MMP‐1, MMP‐3, MMP‐9, and MMP‐14 share a very similar overall fold in their catalytic domains (Fig. 5A). However, sequence differences in their substrate‐binding regions, such as the S‐loop and the specificity loop, which are also involved in binding to N‐TIMP2, can modulate the selectivity of these enzymes (Fig. 5B) [28, 29, 30, 31, 32]. To investigate the selectivity of the N‐TIMP2_MUT_ variant from a structural perspective, we employed molecular modeling and energy minimization. We aimed to predict specific changes in molecular contacts, attributable to individual mutations of N‐TIMP2_MUT_, that may contribute to its enhanced selectivity. The kinetics results demonstrated only a modest reduction of ~ 21‐fold in MMP‐9 inhibition by N‐TIMP2_MUT_ vs. N‐TIMP2_WT_, compared to ~ 180‐fold loss of MMP‐3 inhibition and a much larger > 2500‐fold loss of inhibition of MMP‐1. We therefore modeled and compared complexes of N‐TIMP2_MUT_ with these three MMPs to explore a potential structural explanation for its selectivity. We note, however, that our structural analysis does not explicitly account for the potential impact of loop dynamics. MMP active‐site loops and binding pockets can adopt multiple conformations, as evidenced by crystal structures and NMR data [33, 34], and such flexibility may also shape inhibitor specificity. Although the mutated N‐TIMP2 positions were chosen for their proximity to the binding interface, it is possible that these substitutions indirectly affect the loop dynamics of the complexes; future studies could provide further insight into how loop mobility contributes to the observed specificity.

**Fig. 6:**
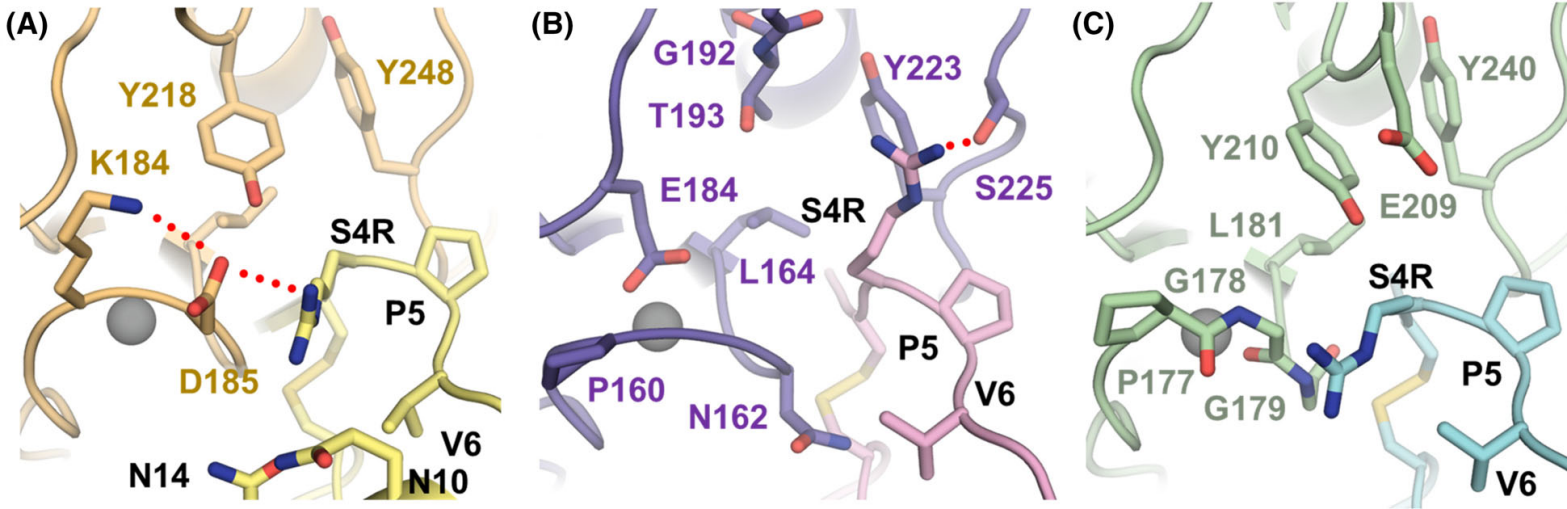
Local environment around the Ser4Arg mutation in complexes of N‐TIMP2_MUT_ with MMP‐9, MMP‐3, and MMP‐1. (A) Modeled complex of MMP‐9 (*light orange*) bound to N‐TIMP2_MUT_ (yellow). (B) Modeled complex of MMP‐3 (*purple*) bound to N‐TIMP2_MUT_ (*pink*). (C) Modeled complex of MMP‐1 (*green*) bound to N‐TIMP2_MUT_ (*cyan*). pymol Molecular Graphics System Version 2.5.2 was used to generate the initial models, followed by the relaxation protocol using YASARA (see Materials and methods).

In the conserved mode of MMP inhibition by TIMPs, the TIMP N‐terminus binds to the MMP catalytic zinc, and TIMP residues 1–4 occupy the primed‐side substrate‐binding subsites of the MMP. These residues are critical for the affinity of TIMPs to MMPs [35], with TIMP residues 2 and 4 being particularly important for selectivity [36]. An examination of the modeled interface around the Ser4Arg substitution suggests that this mutation is important for the selectivity of N‐TIMP2_MUT_ (Fig. 6). In the complex of N‐TIMP2_MUT_ with MMP‐9, Ser4Arg is predicted to form a hydrogen bond network with S‐loop residues Lys184 and Asp185 (Fig. 6A). Since it is predicted that N‐TIMP2_WT_ Ser4 will form a hydrogen bond with either Tyr218 or Asp185, there appears to be no net loss of interaction attributable to the Ser4Arg mutation. In the complex of N‐TIMP2_MUT_ with MMP‐3, Gly192 and Thr193 afford greater space at the interface to accommodate the Arg (a result of Ser4Arg substitution), potentially allowing Arg to penetrate deeper into the substrate cleft and to form a hydrogen bond with Thr193 or with Ser225 in the specificity loop (Fig. 6B). In the complex of MMP‐3 with N‐TIMP2_WT_, Ser4 is predicted to form a hydrogen bond with Glu184 or Gln162 in the backbone of the specificity loop, again implying no net loss of interaction. In contrast, in the complex of N‐TIMP2_MUT_ with MMP‐1, it is predicted that the presence of Tyr210 will restrict the conformation of Arg (a result of Ser4Arg substitution) (Fig. 6C). In the complex of MMP‐1 with N‐TIMP2_WT_, Ser4 is predicted to form a hydrogen bond with either Tyr210 or Gly179. However, in the complex with the N‐TIMP2_MUT_ variant, S‐loop residues Gly178 and Gly179 are not oriented in a manner conducive to hydrogen bonding with Ser4Arg (Fig. 6C), and the consequent loss of interaction may contribute to the variant's loss of affinity for MMP‐1.

The long AB‐loop of N‐TIMP2, which makes contact with the S‐loop of the MMP, harbors two mutations in the N‐TIMP2_MUT_ variant: Ile35Phe and Asn38Asp. Our modeling did not suggest major contributions of these relatively conservative substitutions in the specificity enhancements, despite the known potential of the AB‐loop to influence selectivity [30, 37]. The side chains containing Ile35Phe and Asn38Asp were not predicted to make significantly close contacts with any MMP residues; thus, any contributions of these substitutions to altered specificity may be indirect.

**Fig. 7:**
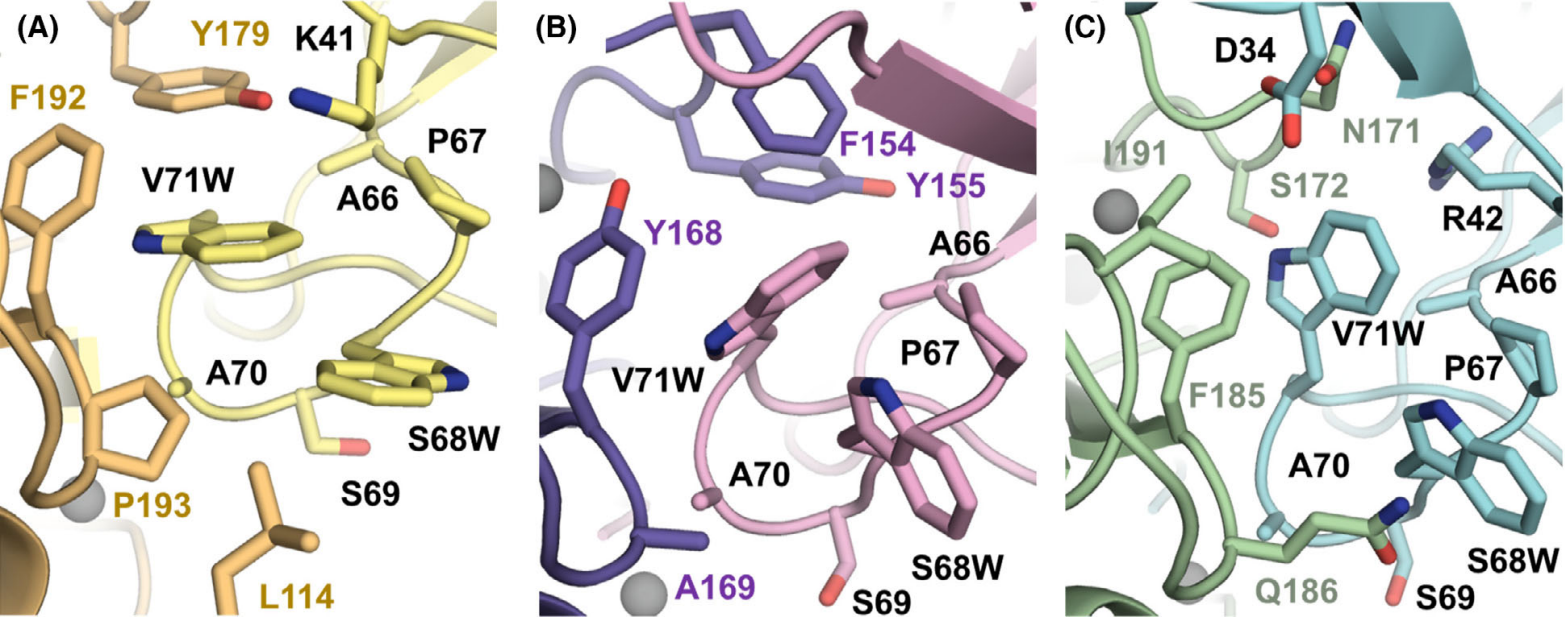
Local environment around the C‐connector loop mutations in complexes of N‐TIMP2_MUT_ with MMP‐9, MMP‐3, and MMP‐1. The region surrounding Ser68Trp and Val71Trp substitutions in N‐TIMP2_MUT_ is shown in modeled complexes of (A) MMP‐9 (*light orange*) bound to N‐TIMP2_MUT_ (*yellow*), (B) MMP‐3 (*purple*) bound to N‐TIMP2_MUT_ (*pink*), and (C) MMP‐1 (*green*) bound to N‐TIMP2_MUT_ (*cyan*). pymol Molecular Graphics System Version 2.5.2 was used to generate the initial models, followed by the relaxation protocol using YASARA (see Materials and methods).

Residues of the TIMP C‐connector loop occupy the non‐primed substrate‐binding subsites of the MMP, interacting with the S‐loop and βIV‐strand of the MMP. Our structural modeling suggests that C‐connector loop substitutions Ser68Trp and Val71Trp may be major contributors to the observed alterations in specificity. In the complex of N‐TIMP2_MUT_ with MMP‐9, the two tryptophan substitutions are predicted to stack well and to pack via aromatic and hydrophobic clustering with MMP‐9 βIV‐strand residues F192 and P193, S‐loop residue Y179, and N‐terminal residue L114 (Fig. 7A). Thus, there appears to be a net gain of interactions in these regions of MMP‐9 compared to the complex with N‐TIMP2_WT_. In the complex of N‐TIMP2_MUT_ with MMP‐3, Val71Trp is predicted to form an aromatic cluster with S‐loop residues Phe154 and Tyr155 and βIV‐strand residue Tyr168 (Fig. 7B). Likewise, Ser68Trp and Pro67 add to strong aromatic and hydrophobic clustering. However, comparisons with the N‐TIMP2_WT_/MMP‐3 complex model reveal that Tyr168, Phe154, and Tyr155 may form a tighter, more organized aromatic cluster with N‐TIMP2_WT_ than with the variant, suggesting that this region may suffer a modest loss of interactions due to the C‐connector loop mutations. In the complex of N‐TIMP2_MUT_ with MMP‐1, the presence of Ile191, Phe185, and Gln186 appears to restrict the positioning of Val71Trp and Ser68Trp (Fig. 7C). Val71Trp is predicted to pack into a pocket formed by Phe185, Ile191, Asn171, and Ser172, while Ser68Trp lacks stabilizing contacts and is constrained by Gln186. In contrast, in the complex of MMP‐1 with N‐TIMP2_WT_, Val71 is predicted to form a hydrophobic π‐contact with Phe185, while Ser68 can form a hydrogen bond with Gln186. Overall, loss of this hydrogen bond in the complex of MMP‐1 with N‐TIMP2_MUT_ and the less optimal packing of the bulky aromatic substitutions may help to explain the loss of inhibition of MMP‐1.

The EF loop of N‐TIMP2 is somewhat removed from the N‐TIMP2‐MMP interface, and its interactions with the bound MMP are therefore limited. Although the EF loop of N‐TIMP2_MUT_ harbored two mutations, His97Ile and Thr99Asp, neither of these residues made direct contact with any of the MMPs in our models. Thus, any impact of these substitutions on selectivity would likely be indirect.

In summary, our molecular modeling results are in qualitative agreement with the experimental kinetics data on the rank ordering of N‐TIMP2_MUT_ interactions with MMPs. Our comparisons suggest that the mutations that are most likely to contribute strongly to the selectivity gain are Ser4Arg, Ser68Trp, and Val71Trp. We identified sequence differences between the three MMPs at positions that interact with the mutated N‐TIMP2 residues that are likely to account for the differential inhibition. However, the additional substitutions within N‐TIMP2_MUT_ may also contribute to the altered specificity in an indirect manner.

## Discussion

Designing selective inhibitors for homologous enzymes poses significant challenges. By leveraging HTS datasets and computational analyses, we successfully addressed the challenge of designing a novel N‐TIMP2 variant with a differential affinity profile: high affinity for MMP‐9, moderate affinity for MMP‐3, and low affinity for MMP‐1. Our method significantly reduces the experimental workload traditionally required for developing selective inhibitors for different targets and offers flexibility in choosing the targets. Once the models are trained, the experimental process can be tailored to identify inhibitors with specificity for various targets. For example, to design a selective N‐TIMP2 inhibitor for MMP‐1, high‐affinity N‐TIMP2 variants for MMP‐1 can be prioritized, while variants with high affinities for MMP‐3 and MMP‐9 can be excluded. By virtue of this adaptability, our method surpasses traditional methods, which often focus solely on screening for high affinity to a single target [26] or rely on labeled target enzymes to isolate desired variants [21].

Since our method relies on computational techniques, it is not limited by the number of enzymes that can be evaluated. Theoretically, it can be expanded to find selective inhibitors among more than three enzymes. This expansion requires only the training of affinity models for additional MMPs and their integration into the existing workflow. For additional MMPs with available HTS data, our framework can efficiently pinpoint selective variants by identifying variants having high affinity for a specific enzyme, while systematically excluding those that bind to undesired targets.

Our method is effective for identifying selective variants, but it may impact the overall affinity of the inhibitor for the target. Indeed, our approach generated the N‐TIMP2_MUT_ variant, which demonstrated enhanced affinity to MMP‐9 compared to MMP‐1 and MMP‐3, but its affinity for MMP‐9 relative to N‐TIMP2_WT_ was reduced 10‐fold. In contrast, Rotenberg *et al*. [26] isolated N‐TIMP2 variants whose affinity for MMP‐9 was similar to that of N‐TIMP2_WT_, while exhibiting selective properties across multiple MMPs (including MMP‐3, MMP‐8, MMP‐10, and MMP‐14). However, these N‐TIMP2 variants exhibited other drawbacks: they contained more mutations than the N‐TIMP2_MUT_ developed in the present study; they did not achieve selectivity against MMP‐1; and despite starting from an N‐TIMP2 variant with selective affinity, they required additional rounds of library generation and screening. Our approach presented here achieved comparable selectivity with the incorporation of fewer mutations, highlighting the potential for balancing selectivity and affinity through computational refinement.

Another limitation of our method is its reliance on HTS data that are derived from deep mutational scanning (DMS) assays for predicting N‐TIMP2 variants. Such datasets require specialized laboratories to generate the necessary data, which can be a significant obstacle in this type of research. Recently, however, databases like MaveDB [38] have made DMS data accessible. An additional limitation is that our predictions are restricted to the mutated positions included in the dataset, limiting the ability to assess the effects of mutations in other positions. For instance, the study of Rotenberg *et al*. [26] identified three new positions (N14, S69, A70) that influence the affinity of N‐TIMP2 for MMPs, but our models cannot predict their impact without HTS data from a library specifically designed for MMP‐9 binding. These unexplored positions may have significant effects that remain unaccounted for.

Future work should focus on incorporating additional conformational characteristics into the models and exploring advanced ML techniques, such as natural language processing, to enhance model performance. Recent breakthroughs in protein language models and structure prediction may further improve the prediction performance of our models and reduce some of the need for experimental HTS data [39, 40]. Expanding mutation libraries and applying this methodology to other inhibitors and homologous enzyme systems will further validate and improve the utility of our method.

In conclusion, we demonstrated the feasibility of using computational approaches combined with HTS data to design selective inhibitors for homologous enzymes. The flexibility and scalability of our method make it a valuable tool for streamlining the development of selective inhibitors. By addressing current limitations and leveraging advances in computational biology, this pipeline holds great potential for transforming inhibitor design and accelerating therapeutic discovery.

## Materials and methods

### Predicting N‐TIMP2 variants with mutations in seven positions and high affinity for MMP‐9

The N‐TIMP2/MMP‐9 model, previously developed in our lab [27], was designed to predict the binding affinity of N‐TIMP2 variants to MMP‐9. It achieved high predictive performance, with Pearson's correlations of > 0.8 in cross‐validation and −0.545 in predicting absolute affinities. Here, we used the model trained on HTS data for the N‐TIMP2_Lib_ (named *MMP9_model.h5* in the repository). To identify high‐affinity variants, we computationally generated all 19^7^ possible sequences with mutations in each of seven key positions. For computational feasibility, we processed the variants in batches, with all possible mutations generated at five mutable positions while keeping the remaining two mutated positions fixed, yielding 19^5^ variants per batch. We filtered each batch to retain only variants containing mutations at all seven positions. From the filtered variants in each batch, we selected the five variants with the highest predicted affinity and selected the top five variants across all batches as high‐affinity candidates (Table 1).

### Processing HTS data for MMP‐1 and MMP‐3 to train our ML models

We trained our ML models on two HTS datasets of a yeast‐surface‐display N‐TIMP2 saturation mutagenesis library, which were sorted and fractioned according to affinity to MMP‐1 and MMP‐3, as described previously [23]. This library, which was designed to contain single mutations at seven key positions (but also contains double and triple mutations, Table 1), was sorted into library fractions based on low and high affinities for MMP‐1 and MMP‐3, which we denote as Gate_T_S_ where T indicates the target protein (i.e., MMP‐1 or MMP‐3), and S indicates the library fraction that includes low or high binding to the target by N‐TIMP2 variants. We denote the original unsorted library population as Gate_PreSort_. In a previous study from our group [23], each library fraction was sequenced with forward and reverse primers for full coverage of the N‐TIMP2 gene. Then, the paired‐end reads were merged into a single sequence using the ‘fast length adjustment of short reads’ (flash) software [41]. In the current study, we analyzed these merged N‐TIMP2 sequences.

For each sequence, we counted the occurrences of each N‐TIMP2 variant in the gate, and calculated the frequency of each variant mut_*j*_ at Gate_T_S_ as follows:

(1) $$f_{{\mathrm{mut}}_{j\left({\text{Gate}}_{T\_S}\right)}}=\frac{\#\text{reads}\;{\mathrm{mut}}_{j\left({\text{Gate}}_{T\_S}\right)}}{\sum_{j=1}^{n}\#\text{reads}\;{\mathrm{mut}}_{j\left({\text{Gate}}_{T\_S}\right)}}$$

where $\#\text{reads}\;{\mathrm{mut}}_{j\left({\text{Gate}}_{T\_S}\right)}$ is the number of reads for variant mut_*j*_ in Gate_T_S_ and $\sum_{j=1}^{n}\#\text{reads}\;{\mathrm{mut}}_{j\left({\text{Gate}}_{T\_S}\right)}$ is the total number of reads for all variants in Gate_T_S_.

Thereafter, to compare the frequencies of each variant to that of the N‐TIMP2_WT_ in the same gate, we calculated the normalized frequency (NF), ${\mathrm{NF}}_{{\mathrm{mut}}_{i\left({\text{Gate}}_{T\_S}\right)}}$, as follows:

(2) $${\mathrm{NF}}_{{\mathrm{mut}}_{j\left({\text{Gate}}_{T\_S}\right)}}=\frac{f_{{\mathrm{mut}}_{j\left({\text{Gate}}_{T\_S}\right)}}}{f_{\mathrm{WT}\left({\text{Gate}}_{T\_s}\right)}}.$$

Based on the NFs, we calculated the ERs of each variant in the gate as follows:

(3) $${\mathrm{ER}}_{{\mathrm{mut}}_{j}\left({\text{Gate}}_{p\_s}\right)}=\frac{{\mathrm{NF}}_{{\mathrm{mut}}_{j}\;\left({\text{Gate}}_{T\_S}\right)}}{{\mathrm{NF}}_{{\mathrm{mut}}_{j}\;\left({\text{Gate}}_{\text{PreSort}}\right)}}$$

We then applied log transformation to base 2 (log_2_) and used the resulting log_2_ ER values as labels for training our ML model.

### Dataset

From the HTS dataset of the full‐length sequences, we filtered out variants with mutations at positions outside the seven targeted positions (4, 35, 38, 68, 71, 97, 99) and those with sequences that were shorter than the N‐TIMP2_WT_ gene. The final dataset included the amino‐acid sequences, log_2_ ER values, and the read count of each variant in each Gate_T_S_ (library fraction). The sequences represent seven highly tolerant binding interface positions, resulting in a total of 4882 N‐TIMP2 variants (Table 2).

### ML model architecture

**Fig. 8:**
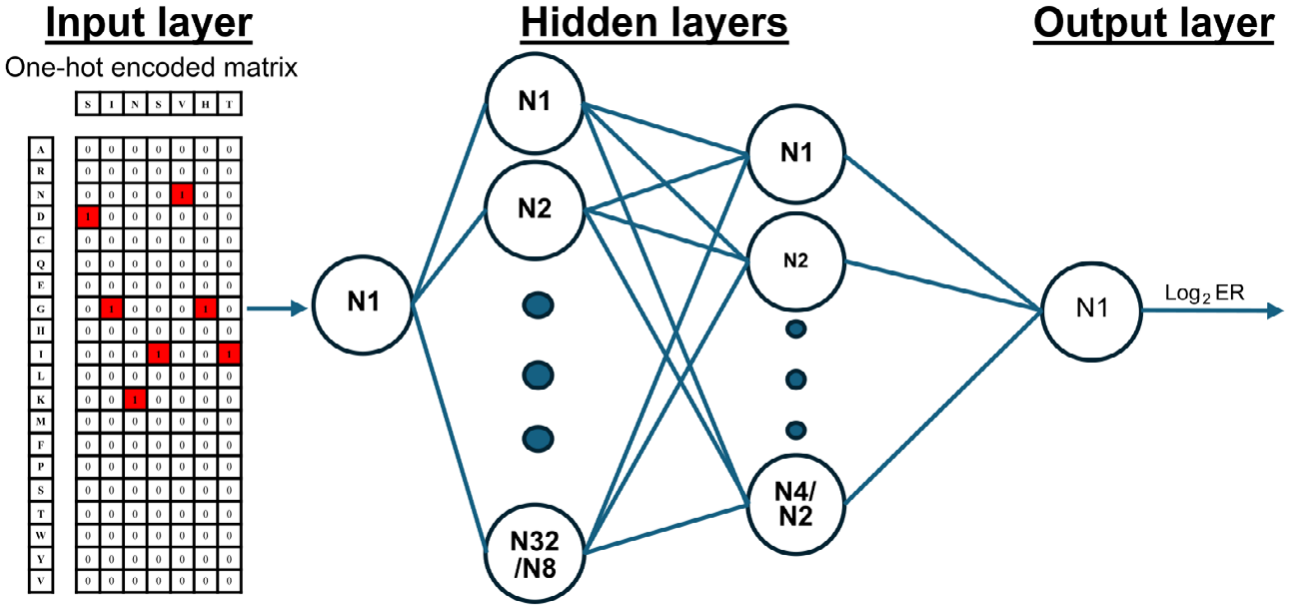
Architecture of the model. The input is a one‐hot encoded matrix of seven binding residues. The model consists of two fully connected layers (FCLs), with a rectified linear unit (ReLU) activation function and a dropout layer (DLR). In the ‘Lib’ architecture: FCL1 = 32 neurons, DLR1 = 20%, FCL2 = 4 neurons, DLR2 = 10%. In the ‘Ala’ architecture: FCL1 = 8 neurons, DLR1 = 0%; FCL2 = 2 neurons, DLR2 = 30%. The output layer is a single neuron with a linear activation function.

We trained four models, each predicting the log_2_ ERs of MMP‐1 or MMP‐3 in the low or high gate. We one‐hot encoded the input sequence of each variant, resulting in a matrix of dimensions 20 × 7 = 140. We flattened the matrices and used them as input to a multi‐layered perceptron network. Based on the N‐TIMP2/MMP‐9 model [27], we tested two architectures (referred to in the original study as ‘Lib’ and ‘Ala’) and selected the one with the best performance in the hyper‐parameter search for each model (Fig. 8).

### Hyper‐parameter search and evaluation

To select the best hyper‐parameters for each model, we sorted the variants in each dataset by the sum of their read counts in Gate_PreSort_ and Gate_T_S_. We selected the top 10% in the total read count to be the test set, and the next 10% as the validation set to ensure high quality and reliable datasets for model evaluation and hyper‐parameter tuning. We tested 360 possible hyper‐parameter combinations across the two architectures (‘Lib’ and ‘Ala’) using the search space that was previously defined in the N‐TIMP2/MMP‐9 model optimization [27] (Table 4). For sample weighting, which is used to assign variable weights to different data points in model training, we used the log_2_ sum of read counts in Gate_PreSort_ and Gate_T_S_, prioritizing variants with larger statistical samples. To enhance model robustness, for each combination we employed the random‐ensemble‐initialization approach, that is, training 10 models with different randomly initialized weights and training batches, as was utilized successfully in the N‐TIMP2/MMP‐9 model [27]. The final prediction output is the average of the 10 trained models. We evaluated each combination by calculating the Pearson's correlation (Eqn 4) between predicted and experimental log_2_ ER values of the validation set. We chose the hyper‐parameter values that lead to the highest Pearson correlation on the validation set of each model separately.

(4) $$\text{Pearson}=\frac{\sum \left(x_{i}-\bar{x}\right)\left(y_{i}-\bar{y}\right)}{\sqrt{\sum {\left(x_{i}-\bar{x}\right)}^{2}\sum {\left(y_{i}-\bar{y}\right)}^{2}}}$$

where $x_{i},y_{i}$ are the value of the predicted/experimental ER and $\bar{x},\bar{y}$ mean of the predicted/experimental ER.

**Table 4:** Hyper‐parameter search range and optimal values. DLR, dropout layer; FCL, fully connected layers.

Hyper‐parameter	Search range	N‐TIMP2/MMP‐1_Low_	N‐TIMP2/MMP‐1_High_	N‐TIMP2/MMP‐3_Low_	N‐TIMP2/MMP‐3_High_
Batch size	{2, 4, 8, 16, 32, 64}	4	8	2	8
Number of epochs	{10, 20, 30, 40, 50}	30	50	40	40
Optimizer learning rate	{10^−4^, 5 × 10^−4^, 10^−3^, 5 × 10^−3^, 10^−2^, 3 × 10^−2^}	0.005	0.001	0.001	0.005
Architecture	{‘Ala’^a^, ‘Lib’^b^}	‘Ala’	‘Lib’	‘Lib’	‘Ala’
Sample weight	{‘no‐weight’, ‘with‐weight’}	‘no‐weight’	‘no‐weight’	‘no‐weight’	‘with‐weight’
Pearson^c^ (for the validation set)	–	0.754	0.363	0.710	0.698
Pearson^c^ (for the test set)	–	0.864	0.64	0.88	0.602

^a^ Architecture: FCL1 = 32 neurons, DLR1 = 20%, FCL2 = 4 neurons, DLR2 = 10%.

^b^ Architecture: FCL1 = 8 neurons, DLR1 = 0%; FCL2 = 2 neurons, DLR2 = 30%.

^c^ Pearson: Pearson correlation was used to determine the linear correlation between the predicted and experimental enrichment ratios (ERs) (Eqn 4; see Materials and methods).

### Independent evaluation of our ML models

To independently evaluate model performance, we obtained *K*_*i*_ values from previous studies. The resultant set included *K*_*i*_ values for complexes of N‐TIMP2_WT_ and N‐TIMP2 variants with MMP‐1 (15 *K*_*i*_ values) and with MMP‐3 (9 *K*_*i*_ values) (Fig. 2). *K*_*i*_ values for all variants were determined using the same method as described under ‘Catalytic activity and inhibition assays’ below. To normalize *K*_*i*_ values and minimize inter‐study variability, we calculated the log_2_ ratio of each variant's *K*_*i*_ to the *K*_*i*_ of the purified N‐TIMP2_WT_ within the same study. Next, we trained a model on the entire dataset, excluding the purified variants to avoid a biased evaluation. Finally, we calculated the Pearson correlation between the predicted log_2_ ER and the normalized log_2_ *K*_*i*_.

### Production and purification of soluble proteins

For the experiments performed to generate the data, we used the catalytic domains of MMP‐1, MMP‐3, and MMP‐9. We used established protocols for expression and purification for the catalytic domains of MMP‐1 and MMP‐3 (designated MMP‐1_CAT_ and MMP‐3_CAT_) [37, 42]. For MMP‐9, we expressed and purified the human MMP9 catalytic domain (MMP‐9_CAT_), without the fibronectin‐like domain (residues 107–215 and 391–443) [43], according to a previously described protocol, with some modifications [44]. We started the procedure with expression of the gene in Bl21(DE3) pLysS *Escherichia coli* cells in a pET28 vector (with an N‐terminal 6 × His tag) and induction with 1 mm isopropyl β‐d‐1‐thiogalactopyranoside overnight at 30 °C. We purified the enzyme by nickel affinity chromatography, followed by anion‐exchange chromatography and size‐exclusion chromatography. We determined protein concentrations by measuring UV–Visible absorbance at 280 nm, using extinction coefficients (ε280) of 25 440, 28 420, and 33 920 m ^−1^·cm^−1^ for MMP‐1, MMP‐3, and MMP‐9, respectively, on a NanoDrop Spectrophotometer (Thermo Scientific, Waltham, MA, USA). Finally, we confirmed the purity of the proteins by SDS/PAGE analysis. The concentrations of MMP‐1, MMP‐3, and MMP‐9 were quantified by active site titration using N‐TIMP2_WT_, which binds all enzymes with sufficiently strong affinity to ensure quantitative stoichiometric complex formation at the enzyme concentrations employed (10 nm) [45].

We produced soluble N‐TIMP2_WT_ and N‐TIMP2_MUT_ proteins in *Pichia pastoris* strain X‐33 using pPICZα (Invitrogen, Waltham, CA, USA), as previously described with some modifications [21]. Briefly, we cloned N‐TIMP2_WT_ and N‐TIMP2_MUT_ into the pPICZαA vector for expression in Pichia* pastoris* strain X‐33. Both proteins carried c‐Myc and 6 × His tags at the C‐terminus for protein detection and purification, respectively. We purified the proteins from the yeast growth medium with nickel nitrilotriacetic acid‐Sepharose beads (Invitrogen) that were equilibrated in a mixture of 50 mm Tris, pH 7.5, 300 mm NaCl, and 10 mm imidazole. The elution buffer was 50 mm Tris, pH 7.5, 300 mm NaCl. We further purified the proteins by gel filtration using a Superdex 75 column (GE Healthcare, Pittsburgh, PA, USA) that was equilibrated in 50 mm Tris, pH 7.5, 300 mm NaCl, and 5 mm CaCl_2_ at a flow rate of 0.8 mL·min^−1^ on an ÄKTA pure instrument (GE Healthcare). We used SDS/PAGE and mass spectrometry analysis (Ilse Katz Institute for Nanoscale Science and Technology, BGU, Israel) to verify protein sizes. We determined protein concentrations using UV–Vis absorbance at 280 nm on a NanoDrop Spectrophotometer (ε280 of 13 325 m ^−1^·cm^−1^ for N‐TIMP2_WT_ and N‐TIMP2_MUT_). Yields for all purified proteins ranged from 2 to 21 mg·L^−1^.

### Catalytic activity and inhibition assays

We performed catalytic activity and inhibition assays as previously described with minor modifications [24]. We tested N‐TIMP2_WT_ and its variant N‐TIMP2_MUT_ for inhibitory activity by incubating them with the three different MMPs at the following concentrations: 0.325 nm MMP‐1 with 0.325 to 16 nm N‐TIMP2_WT_ or with 0.1 to 250 nm of N‐TIMP2_MUT_; 0.325 nm MMP‐3 with 0.325–16 nm N‐TIMP2_WT_ or with 0.1 to 250 nm of N‐TIMP2_WT_; or 0.325 nm MMP‐9 with 0.325 to 16 nm N‐TIMP2_WT_ or N‐TIMP2_MUT_. We performed all incubations in TCNB buffer (50 mm Tris, pH 7.5, 100 mm NaCl, 5 mm CaCl_2_, and 0.05% Brij) for 1 h at 37 °C. After incubation, we added the fluorogenic substrate Mca‐Pro‐Leu‐Gly‐Leu‐Dpa‐Ala‐Arg‐NH_2_·TFA [Mca = (7‐methoxycoumarin‐4‐yl)acetyl, Dpa = *N*‐3‐(2,4‐dinitrophenyl)‐l‐2,3‐diaminopropionyl and TFA = trifluoroacetic acid] (Merck Millipore, Burlington, MA, USA) at a final concentration of 7.5 μm for all MMPs. We used a Synergy 2 plate reader (BioTek, Winooski, VT, USA) to monitor the fluorescence at 37 °C using 340/30 excitation and 400/30 emission filters. We tracked the fluorescence for 60 min and calculated initial rates from the linear portion of fluorescence increase caused by substrate cleavage. To verify that equilibrium was achieved, before data collection we tested a range of preincubation times (up to 120 min) and confirmed that 60 min yielded indistinguishable initial rates; extending the preincubation further did not change slope, indicating the system had reached equilibrium for all MMP–N‐TIMP2 pairs. We globally fitted the data by multiple regression to Morrison's tight binding inhibition equation (Eqn 5) using graphpad prism 7 (San Diego, CA, USA). To calculate the inhibition constant, *K*_*i*_, we plotted the initial velocities against different concentrations of the inhibitors. The reported *K*_*i*_ values are the means of three independent experiments ± standard deviation. In the calculations, we used *K*_*m*_ values of 3.607 ± 0.598 μm for MMP‐1, 3.771 ± 0.428 μm for MMP‐3, and 4.75 ± 0.528 μm for MMP‐9, as determined using Mca‐Pro‐Leu‐Gly‐Leu‐Dpa‐Ala‐Arg‐NH_2_·TFA from at least three Michaelis–Menten kinetic experiments performed in our laboratory.

(5) $$\frac{V_{i}}{V_{0}}=\frac{1-\left(\left[E\right]+\left[I\right]+K_{i}^{\mathrm{app}}\right)-\sqrt{{\left(\left[E\right]+\left[I\right]+K_{i}^{\mathrm{app}}\right)}^{2}-4\left[E\right]\left[I\right]}}{2\left[E\right]}$$

where *V*_*i*_ and *V*_*0*_ are the enzyme (MMP) velocities in the presence and absence of the relevant N‐TIMP2 inhibitor, respectively; *E* and *I* are the concentrations of enzyme and inhibitor, respectively; *K*_*m*_ is the Michaelis–Menten constant; and $K_{i}^{\mathrm{app}}$ is the apparent inhibition constant, which is given by: $K_{i}^{\mathrm{app}}=K_{i}\left(1+\frac{\left[S\right]}{K_{m}}\right)$, where *S* is the substrate concentration. By including both [S] and *K*_*m*_ as fixed parameters in the Prism global fit of Eqn (5), we obtain the intrinsic *K*_*i*_ value.

To evaluate the specificity of N‐TIMP2_WT_ or N‐TIMP2_MUT_ for the MMPs, we calculated the fold change (*K*_*i*_ fold) in the N‐TIMP2 inhibition constant for MMP‐9 relative to the fold changes for MMP‐1 and MMP‐3.

### Molecular modeling analysis

The model of MMP‐9/N‐TIMP2_WT_ was previously constructed and utilized by our group [46], by superposing the MMP‐9 chain from PDB ID: 4JIJ [47] onto the MMP‐14 chain of PDB ID: 1BUV [48]. To create the MMP‐1/N‐TIMP2_WT_, we superposed the N‐TIMP2 chain from the existing model onto the TIMP1 chain of PDB ID: 2J0T [49]. Similarly, the MMP‐3/N‐TIMP2_WT_ model aligned the N‐TIMP2 chain onto the TIMP‐1 chain of PDB ID: 1UEA [50].

To generate the MMP/N‐TIMP2_MUT_ complexes, we introduced mutations using pymol (Schrodinger, LLC, New York, NY, USA. The PyMOL Molecular Graphics System, Version 2.5.2). The rotamers of the mutated side chains were adjusted to minimize clashes. All models were subjected to identical energy minimization protocols [51], which included 500 ps of minimization with YASARA [52] using the YASARA2 forcefield in a cubic simulation box extending 10 Å from the protein. The simulation box was filled with TIP3P water of density 0.997 g·L^−1^ and Na^+^/Cl^−^ counterions at 0.9%, at a temperature of 298K and a pH of 7.4. The simulation integration timestep was 2 fs, and atomic coordinates were saved as a simulation frame every 25 ps. We plotted the global energy of these frames to ensure energetic convergence of the relaxation, and chose representative frames for structural comparisons.

## Conflict of interest

The authors declare no conflict of interest.

## Author contributions

ESR, YO, and NP designed the research; DSG, RM, GS, and MC performed the research; DSG, RM, GS, MC, ESR, YO, and NP analyzed the data; DSG, MC, ESR, YO, and NP wrote the paper.

## Data Availability

HTS data used in this study are publicly available in NCBI Gene Expression Omnibus (GEO) repository under accession number GSE290918: https://www.ncbi.nlm.nih.gov/geo/query/acc.cgi?acc=GSE290918. The code trained models and processed datasets are available at https://github.com/OrensteinLab/N‐TIMP2‐‐MMP‐selectivity. The protein structures used in this study are publicly available from the Protein Data Bank (PDB). The following accession codes correspond to the structural models analyzed in this manuscript: 1BUV, 4JIJ, 1UEA, and 2J0T.
